# Risk of Type 2 Diabetes Among the Pakistani Population: Results of a Cross-sectional Survey

**DOI:** 10.7759/cureus.3144

**Published:** 2018-08-14

**Authors:** Fauzia H Mohammad, Kashmira Nanji

**Affiliations:** 1 Family Medicine, The Aga Khan University, Karachi, PAK; 2 Epidemiology and Public Health, The Aga Khan University, Karachi, PAK

**Keywords:** t2dm, factors, predictors, pakistan

## Abstract

Introduction

Diabetes is a global issue. The aim of this study was to identify the high-risk population and factors associated with the future development of type 2 diabetes mellitus (T2DM).

Methods

A cross-sectional study was conducted in the Family Medicine clinics of a tertiary care hospital in Karachi, Pakistan, from July 2016 to February 2017. Patients or patient care attendants aged 21 to 85 years visiting the clinics were included. Individuals with known diabetes or with serious comorbid conditions were excluded. A total of 600 participants were consecutively approached. QDiabetes (an online diabetes risk calculator) was used to measure the risk of developing T2DM. IBM SPSS Statistics for Windows, Version 19.0 (IBM Corp., Armonk, NY) was used for data analysis.

Results

Information from a total of 522 participants was included in the final analysis. Roughly 53% of them were between 25 and 44 years of age. There was a predominance of females (63%). Thirty-eight percent of patients were at high-risk. The factors associated with high risk of T2DM were as follows: age 65 years or greater (Relative risk [RR]: 5.81; 95% confidence interval [CI]: 2.01 to 16.76); female (RR: 1.86; 95% CI: 1.05 to 3.28, p = 0.03); a past history of hypertension (RR: 5.11; 95% CI: 2.49 to 10.49); a family history of diabetes (RR: 9.76; 95% CI: 5.49 to 13.35).

Conclusion

Controlling glucose levels and preventing hyperglycemia is a challenging task due to the increasing trend of a sedentary lifestyle and changes in dietary patterns. Counseling should be provided to caregivers and high-risk patients on the prevention of T2DM including lifestyle modifications.

## Introduction

Diabetes is a global issue. Type 2 diabetes mellitus (T2DM) is the most common form of diabetes. Globally, 80% to 90% of diabetes cases are T2DM [1]. According to the International Diabetes Federation (IDF), it is estimated that almost 415 million people had diabetes worldwide in 2015, and by the year 2040, the number is expected to rise to 642 million with more than 80% of these cases in developing countries [2]. The number of deaths due to diabetes was five million in 2015 [2].

The IDF has projected that by the year 2025, 11.5 million people in Pakistan will be living with diabetes, placing Pakistan fifth on the IDF ranking of diabetes populations [3,4]. The increasing prevalence of diabetes is mainly attributed to population growth, aging, urbanization, and the increasing prevalence of obesity and physical inactivity [5-7].

Almost 200 million people with diabetes are undiagnosed and are, therefore, at a greater risk of developing complications such as kidney failure, blindness, amputations, and stroke. These complications may be prevented by identifying the high-risk population [8,9]. In many cases, T2DM can be prevented by adopting a healthy lifestyle.

Therefore, identifying high-risk populations (obese patients, positive family history of diabetes, etc.) is imperative in saving lives and can assist in preventing or significantly delay devastating diabetes-related complications [10]. The aim of this study was to identify the high-risk population and the factors associated with future development of T2DM.

## Materials and methods

A cross-sectional study was conducted in Family Medicine clinics affiliated with a tertiary care teaching hospital in the private sector in Karachi, Pakistan, from July 2016 to February 2017. These clinics were included because they cater to the health needs of people from different socioeconomic strata, providing the study a sample from a diverse population.

Patients or their attendants, 21 to 85 years of age, were invited to participate in the study. However, individuals with known diabetes or suffering from serious comorbid conditions such as cancer, advanced heart failure, or other comorbidities, were excluded. A total of 600 participants were approached to take part in this study. Written informed consent was obtained from all participants, and the study was conducted in accordance with the Declaration of Helsinki.

A previously validated structured questionnaire was used for data collection. The first section was composed of demographic details while the second section included the online calculator QDiabetes [11] to measure the absolute risk of developing T2DM. It has been validated and is widely used in various studies. This online calculator uses factors such as age, sex, smoking status, family history of diabetes, history of cardiovascular disease, history of hypertension, and body mass index (BMI) to calculate the risk of developing T2DM. Before commencement of the study, data collectors were trained for obtaining consent and filling up of the forms.

We have categorized relative risk (RR) into two groups. An RR less than one was labeled as low while an RR of more than one was labeled as high-risk of developing T2DM.

Asian BMI cutoffs were used in this study. The categories of BMI for Asians are as follows: less than 18.5 kg/m^2^ (underweight); 18.5 to 23 kg/m^2^ (normal); 23 to 27.5 kg/m^2^ (overweight); and 27.5 kg/m^2^ or higher (obese).

Data were entered and analyzed in IBM SPSS Statistics for Windows, Version 19.0 (IBM Corp., Armonk, NY). The analysis was performed in two stages (i.e., descriptive and inferential). The frequencies and proportions of all the variables were reported in the descriptive analysis. A Pearson chi-square test was applied to compare low-risk and high-risk individuals. A multivariable Cox regression analysis was used to study the independent association of variables with the presence of a high-risk of diabetes. The RR with 95% confidence intervals (CIs) was estimated.

## Results

A total of 600 participants were approached. Of those, 522 gave consent and were included in the final analysis, yielding a response rate of 87%. Table 1 presents the baseline characteristics of the study participants. About 53% of the participants were between 25 and 44 years of age. There was a predominance of women in the study sample (63%). Slightly more than two-fifths (42%) of the participants had a family history of diabetes while 24% had a history of hypertension. About 36% of the participants were obese, and 26% were morbidly obese. Approximately 38% of the participants were calculated to have a high risk of diabetes in the next 10 years.

**Table 1:** Baseline characteristics of study participants (n = 522).

Variables	Frequency	Percentage
Age		
25-44 years	279	53
45-64 years	191	36
>65 years	52	10
Gender		
Male	192	37
Female	330	63
Smoking		
Yes	34	6
Family History of Diabetes		
Yes	220	42
Past History of Hypertension		
Yes	124	23.7
Past History of Cerebrovascular Accident (CVA)		
Yes	7	1.3
Body Mass Index (BMI)		
Underweight	16	3
Normal weight	105	20
Overweight	76	14
Obese	325	63
Future Risk of Diabetes		
Low risk	325	62
High risk	197	38

Seventy-four percent of men and 55% of women were at a low risk of developing diabetes in the future (Table 2). A past history of hypertension among men (31%) and women (35%) was predictive of being at high risk.

**Table 2:** Gender-specific risk of diabetes among study participants (n = 522).

Variables	Men		Women	
Parameter n = 522	Low Risk (n = 143)	High Risk (n = 49)	Low Risk (n = 182)	High Risk (n = 148)
Age				
25-44 years (%)	72 (50.3)	30 (61.2)	98 (53.8)	79 (53.4)
45-64 years (%)	51 (35.7)	17 (34.7)	61 (33.5)	62 (41.9)
>65 years (%)​​​​​​​	20 (14.0)	2 (4.1)	23 (12.6)	7 (4.7)
P value	0.13		0.028	
Current Smokers				
Yes (%)​​​​​​​	22 (15.4)	10 (20.4)	1 (0.5)	1 (0.7)
P value	0.41		0.883	
Family History of Diabetes				
Yes (%)​​​​​​​	29 (20.3)	32 (65.3)	61 (33.5)	98 (66.2)
P value	0.000		0.000	
Past History of Hypertension				
Yes (%)​​​​​​​	26 (18.2)	15 (30.6)	32 (17.6)	51 (34.5)
P value	0.06		0.000	
Past History of Cerebrovascular Accident (CVA)				
Yes (%)​​​​​​​	----	5 (10.6)	2 (1.1)	---
P value	0.185		0.201	
Body Mass Index (BMI)				
Under weight (%)​​​​​​​	8 (5.6)	---	8 (4.4)	---
Normal weight (%)​​​​​​​	54 (37.8)	---	51 (28.0)	---
Overweight (%)​​​​​​​	28 (19.6)	1 (2.0)	47 (25.8)	---
Obese (%)​​​​​​​	53 (37)	48 (98)	76 (42)	148 (100)
P value	0.000		0.000	

Participants who were 65 years or older (RR: 5.81; 95% CI: 2.01 to 16.76), were women (RR: 1.86; 95% CI: 1.05 to 3.28), or had a past history of hypertension (RR: 5.11; 95% CI: 2.49 to 10.49) were significantly more likely to have a predicted high risk of future T2DM (Table 3).

**Table 3:** Factors associated with high risk of diabetes among study participants. CI: Confidence interval; BMI: Body mass index.

Variables	Unadjusted Relative Risk (95% CI)	Adjusted Relative Risk (95% CI)	P value
Age 45-64 years	3.06 (1.43-6.53)	4.90 (1.76-13.61)	0.004
Age ≥ 65 years	3.37 (1.55-7.30)	5.81 (2.01-16.76)	
Female	2.37 (1.60-3.50)	1.86 (1.05-3.28)	0.03
Past history of hypertension	2.31 (1.53-3.49)	5.11 (2.49-10.49)	<0.001
Family history of diabetes	5.06 (3.45-7.43)	9.76 (5.49- 13.35)	<0.001
High-risk BMI	5.26 (4.93-13.99)	8.97 (4.12-12.51)	<0.001

## Discussion

In our study, 38% of the study participants were at a high risk of developing diabetes. Moreover, factors such as being older than 65 years of age, female, obese, having a family history of diabetes, or a personal history of hypertension are predictors of developing diabetes in future. Our study results are congruent with previous studies, and, hence, the control and prevention of these factors are essential, particularly among high-risk patients, to prevent the future occurrence of T2DM in this population.

Several studies suggest that hypertension is more common in people with diabetes than in the general population [12,13]. This is consistent with our findings, and suggests that a history of hypertension is highly associated with developing diabetes in the future (RRadj: 5.11; 95% CI: 2.49 to 10.49).

A 10-year cohort study revealed that, when compared with their same-sex peers, patients with a BMI greater than 25 were approximately 20 times more likely to develop diabetes (RR: 17.0 for women; RR: 23.4 for men) [14]. This is consistent with the results of the current study where having a BMI of more than 25 increased the risk of developing diabetes approximately nine-fold.

Evidence suggests that smoking increases the risk of diabetes many times [15,16]. A meta-analysis conducted on 88 studies concluded that there was a dose-response relationship for current smoking and diabetes risk: the RRs were 1.34 for moderate and 1.57 for heavy smokers [16]. Public health efforts to reduce smoking could have a substantial effect on the worldwide burden of T2DM. In this study, 0.6% of the respondents were smokers. Since smoking is generally considered as socially unacceptable, there is a possibility of under-reporting by the study participants.

A history of cardiovascular disease (CVD) has been reported by several studies to be associated with a higher risk of developing diabetes [17,18]. In this study, however, a history of CVD did not have a significant association with the calculated future risk of diabetes. This may be because only a small number of study participants had CVD (1.3%).

Evidence from Pakistan suggests that women have a higher risk of poor glycemic control resulting in further complications [19,20]. The current study also found that females had a higher risk of developing T2DM (RR: 1.86; 95% CI: 1.05 to 3.28). In Pakistan, the health of women is compromised as they are not empowered to make many of their own decisions and in some cases are not allowed to go outside their homes without a male member of the household [21]. These issues leave them more exposed to a sedentary lifestyle and obesity.

This study had certain limitations. As it was a cross-sectional study, we cannot comment on the causal associations of the factors with the risk of T2DM. Therefore, cohort studies are needed to determine the causality between the factors that increases the risk of DM. In this study, we estimated the risk of future DM from a pre-designed online calculator, which might have not included all the confounders, this may have led to over or underestimation of the study results. Moreover, we did not check glycosylated hemoglobin, the best marker for the identification of individuals with undiagnosed diabetes, as we could not arrange funds for the laboratory work. Moreover, this study was conducted in the primary clinics of a tertiary care hospital, so the results may not be generalizable to the general population.

## Conclusions

Controlling glucose levels and preventing hyperglycemia is a challenging task due to increasing trends towards a sedentary lifestyle and changes in dietary patterns. Nonetheless, this study serves as a basis for future interventional studies to reduce the immense future burden of T2DM. Based on our study findings, we recommend that counseling should be provided to caregivers and high-risk patients regarding T2DM prevention and lifestyle modifications including increased physical activity and smoking cessation. Moreover, nutrition intervention should be tailored according to each patient’s age, lipid levels, and medical conditions.
